# Oxytocin and cortisol in goat milk during early lactation under different housing conditions: a descriptive study

**DOI:** 10.3389/fvets.2026.1820696

**Published:** 2026-04-28

**Authors:** Manja Zupan Šemrov, Maja Peretin, Nina Čebulj-Kadunc

**Affiliations:** 1 Department of Animal Science, Biotechnical Faculty, University of Ljubljana, Ljubljana, Slovenia; 2 Department of Preclinical Sciences, Veterinary Faculty, University of Ljubljana, Ljubljana, Slovenia

**Keywords:** body weight, litter size, positive animal welfare, social environment, stress physiology

## Abstract

This Brief Research Report descriptively characterizes oxytocin (OT) and cortisol (CORT) concentrations in milk of multiparous dairy goats (*n* = 20) during early lactation and explores associations with housing context and early kid growth within a Positive Animal Welfare framework. Does and their kids were housed in two indoor pens (low density, LD; 1.39 m^2^ per doe; high density, HD; 0.89 m^2^ per doe).Milk samples were collected weekly over four consecutive weeks during machine milking and kid suckling. Mean milk OT concentrations were 296.7 ± 133.6 pg/mL during milking and 284.9 ± 119.7 pg/mL during suckling, whereas mean CORT concentrations were 0.86 ± 0.72 and 0.75 ± 0.71 μg/dl, respectively. OT concentrations were highest in week 1 and declined over time, while CORT showed no consistent temporal pattern. Does in LD showed higher OT and lower CORT than HD during both milking (OT: 336 ± 141 vs. 257 ± 116 pg/ml; CORT: 0.72 ± 0.63 vs. 1.00 ± 0.77 μg/dl) and suckling (OT: 309 ± 123 vs. 260 ± 111 pg/ml; CORT: 0.64 ± 0.62 vs. 0.86 ± 0.76 μg/dl). Higher maternal OT and lower CORT were associated with greater kid body weight at 51 days of age, whereas twin-rearing does showed higher CORT and lower kid weight than single-rearing does. These findings demonstrate the feasibility of repeated OT and CORT measurement in goat milk under routine farm conditions, but interpretation is limited by small sample size, lack of pen replication, and incomplete assay validation.

## 1 Introduction

Dairy goat production is an expanding sector of European livestock farming, where productivity and animal welfare must be balanced under commercial conditions (1). Indoor group housing systems are common, and factors such as space allowance and stocking density may influence behavioral regulation and physiological stress responses. Identifying practical physiological indicators measurable under routine farm conditions is therefore relevant within contemporary Positive Animal Welfare (PAW) frameworks (2, 3). Oxytocin (OT) plays a central role in milk ejection in ruminants, being reflexively released during suckling and machine milking (4–6). Beyond its lactational function, OT contributes to maternal behavior and modulation of stress-related processes (7, 8). Cortisol (CORT), the end product of hypothalamic–pituitary–adrenal activation, reflects physiological responses to environmental demands. Interactions between OT and CORT systems suggest that these hormones may jointly reflect adaptive regulation and coping capacity rather than simple positive or negative affective states.

Housing conditions, including stocking density, influence stress physiology in livestock (9), and OT has been proposed as a candidate physiological indicator when interpreted alongside glucocorticoids (10). Although OT dynamics in blood have been widely studied in dairy ruminants, reports quantifying endogenous OT concentrations directly in goat milk remain limited. Milk represents a practical and minimally invasive biological matrix, yet its potential to reflect maternal endocrine state and its association with offspring development are insufficiently explored in dairy goats. Evidence from other species suggests that milk-borne hormones may influence offspring growth and stress regulation (11–13).

Although OT and CORT are well-established components of lactational physiology and stress regulation, their combined interpretation in relation to social environment and maternal state remains insufficiently understood in dairy goats. In particular, while both hormones are known to respond to environmental and social conditions, and to play roles in maternal behavior and offspring development, their measurement in milk as a practical, non-invasive matrix has received limited attention. This is especially relevant under commercial farm conditions, where repeated sampling of blood or other matrices is often not feasible. Furthermore, the potential links between maternal milk hormone concentrations, environmental context, and early offspring development remain poorly explored in goats. Addressing these gaps is important for advancing the use of physiologically grounded, feasible indicators within the framework of positive animal welfare.

This Brief Research Report therefore aimed to descriptively characterize OT and CORT concentrations in goat milk during early lactation under routine farm conditions and to explore descriptive variation across the two housing conditions present on the farm, as well as associations with early kid growth and litter size.

## 2 Material and methods

### 2.1 Animals and housing

The study was conducted from March to May 2016 on a Slovenian commercial dairy goat farm consisting of 55 multiparous Slovenian Alpine does (approximately 2 years of age) and two adult bucks of the same breed. Since spring 2015, goats had been randomly allocated using block randomization to two housing conditions differing in population density: high density (HD) and low density (LD). Of the 55 does, 27 were housed in HD and 26 in LD; 10 per group were randomly selected for milk sampling. The study followed a repeated-measures design, with milk samples collected weekly over four consecutive weeks from the same does under two sampling contexts: machine milking and kid suckling.

Each group was housed in a deep straw bedding pen (25 m^2^; 5 × 5 m) separated by wooden partitions and sharing a roughage rack (0.6 × 5.0 m), providing comparable feeder space per animal. Concentrate feeding was conducted in identical self-closing cribs during milking, minimizing direct competition at the feeder. In addition, the LD group had access between morning and evening milking to an adjacent 12.5 m^2^ area. Effective space allowance was approximately 1.39 m^2^ per doe in LD and 0.83 m^2^ per doe in HD. Visual, auditory, and olfactory contact between groups was maintained. Natural lighting was provided through roof windows; artificial light was used during feeding and morning milking. Barn temperature ranged from 8.0 to 26.7 °C. Two drinkers were available per pen.

Second kidding occurred in March–April 2016. Kids remained with their mothers except during milking and scheduled supervised suckling. No milk replacer was used. One doe neglecting a twin kid was excluded; one triplet litter was analyzed as twins.

Both groups received identical diets. Hay was provided as the main roughage after morning milking. During morning and evening milking, does received 0.65 kg concentrate per milking in self-closing cribs in the milking parlor. The commercial concentrate (formulated for cattle) contained 10.0% crude protein, 2.3% fat, 3.0% fiber, 5.6% ash, and 0.20% Na.

### 2.2 Milk sampling and laboratory procedures

Milk samples were collected from 20 clinically healthy multiparous does (10 per housing condition). Each doe was sampled once weekly over four consecutive weeks during machine milking and once during kid suckling (total *n* = 160 samples). Samples were cooled immediately to 4 °C and transported to the laboratory within 5 h. Milk was centrifuged at 9,000 rpm at 4 °C for 20 min. The supernatant was collected after removal of the fat layer; centrifugation was repeated twice. Supernatants were stored at −20 °C until analysis. Milk oxytocin (OT) concentrations were measured using a commercial ELISA kit (Oxytocin ELISA, DE3117, Demeditec Diagnostics GmbH, Germany; assay range 15.6–1,000 pg/ml; analytical sensitivity 15 pg/ml).

Intra-assay CV ranged from 10.2 to 13.3%; inter-assay CV from 11.8 to 20.9%. Milk cortisol (CORT) concentrations were measured using a commercial ELISA kit (Cortisol Saliva ELISA, RE52611, IBL International GmbH, Germany; assay range 0.015–3 μg/dl; analytical sensitivity 0.003 μg/dl). Intra-assay CV ranged from 3.2 to 6.1%; inter-assay CV from 4.2 to 17%. Parallelism and spike–recovery validation for goat milk were not performed; results are therefore interpreted descriptively. However, milk cortisol has previously been measured in goats and shown to reflect physiological and management-related variation, supporting its use as a biologically relevant, non-invasive indicator (14).

### 2.3 Kid measurements

Kid body weight was recorded within 24 h of birth and at four subsequent time points, including a standardized measurement at 51 days of age.

### 2.4 Statistical analysis

Analyses were performed using SAS 9.4 (SAS Institute Inc., Cary, NC, USA). Distribution of OT and CORT concentrations was evaluated using the UNIVARIATE procedure and did not substantially deviate from normality. Two high CORT values from one doe were retained, as no biological or technical justification for exclusion was identified. Given the absence of pen-level replication, results are primarily presented descriptively. Means, standard deviations, and ranges were calculated for hormone concentrations across weeks, sampling contexts, housing conditions, and litter sizes. Associations between maternal hormone concentrations and kid body weight at 51 days were explored at the doe level using Pearson correlations and linear regression models. Model fit was described using root mean square error (RMSE). A significance threshold of *P* ≤ 0.05 was applied; however, interpretation focuses on effect direction and magnitude given the exploratory design.

## 3 Results

### 3.1 Individual variation in milk oxytocin and cortisol

Milk oxytocin (OT) and cortisol (CORT) concentrations showed substantial individual variation among the 20 does (Table 1). Across all sampling events, mean OT concentrations were 296.7 ± 133.6 pg/mL during machine milking and 284.9 ± 119.7 pg/mL during kid suckling (ranges: 68.2–852.0 and 63.2–592.0 pg/ml, respectively). Mean CORT concentrations were 0.86 ± 0.72 μg/dl during milking and 0.75 ± 0.71 μg/dl during suckling (ranges: 0.16–3.69 and 0.15–3.94 μg/dl, respectively).

**Table 1:** Individual mean (± SD) oxytocin (OT, pg/ml) and cortisol (CORT, μg/dl) concentrations in milk of does (*n* = 20) during machine milking and kid suckling over four consecutive weeks.

Doe	Pen	OT milking (pg/ml)	OT suckling (pg/ml)	CORT milking (μg/dl)	CORT suckling (μg/dl)
1	HD	182.9 ± 25.0	166.9 ± 15.5	0.4 ± 0.3	0.6 ± 0.3
2	HD	303.5 ± 131.3	293.3 ± 126.2	1.3 ± 0.7	0.8 ± 0.4
3	HD	322.5 ± 65.6	322.4 ± 93.6	0.8 ± 0.3	0.8 ± 0.4
4	HD	243.0 ± 68.2	245.1 ± 67.2	0.5 ± 0.3	1.0 ± 0.9
5	HD	305.5 ± 133.1	307.8 ± 128.8	0.5 ± 0.2	0.5 ± 0.5
6	HD	179.0 ± 19.4	190.1 ± 55.3	0.2 ± 0.0	0.2 ± 0.0
7	HD	148.3 ± 77.2	166.8 ± 98.4	2.6 ± 0.6	2.2 ± 1.0
8	HD	238.3 ± 64.1	347.6 ± 112.1	0.6 ± 0.3	0.3 ± 0.1
9	HD	317.3 ± 40.0	281.0 ± 15.6	1.8 ± 0.9	0.7 ± 0.3
10	HD	268.4 ± 55.2	232.4 ± 52.8	0.3 ± 0.1	0.5 ± 0.4
11	LD	293.9 ± 132.4	308.7 ± 178.0	0.9 ± 0.3	1.1 ± 0.5
12	LD	241.7 ± 55.0	246.2 ± 34.5	0.6 ± 0.2	0.6 ± 0.3
13	LD	368.5 ± 95.9	388.2 ± 114.9	1.2 ± 0.7	0.5 ± 0.1
14	LD	486.0 ± 228.7	353.2 ± 133.8	0.6 ± 0.3	0.4 ± 0.0
15	LD	262.9 ± 50.2	446.7 ± 127.7	0.9 ± 0.5	0.5 ± 0.3
16	LD	405.6 ± 116.5	327.1 ± 98.0	0.4 ± 0.2	0.3 ± 0.2
17	LD	396.3 ± 112.8	374.5 ± 69.5	0.3 ± 0.2	0.5 ± 0.1
18	LD	383.3 ± 120.0	338.1 ± 68.5	0.2 ± 0.0	0.3 ± 0.1
19	LD	218.5 ± 47.3	231.7 ± 57.1	1.0 ± 0.4	0.9 ± 0.4

Pen: HD, greater space allowance pen; LD, lower space allowance pen.

### 3.2 Weekly patterns in oxytocin and cortisol concentrations

**Figure 1:**
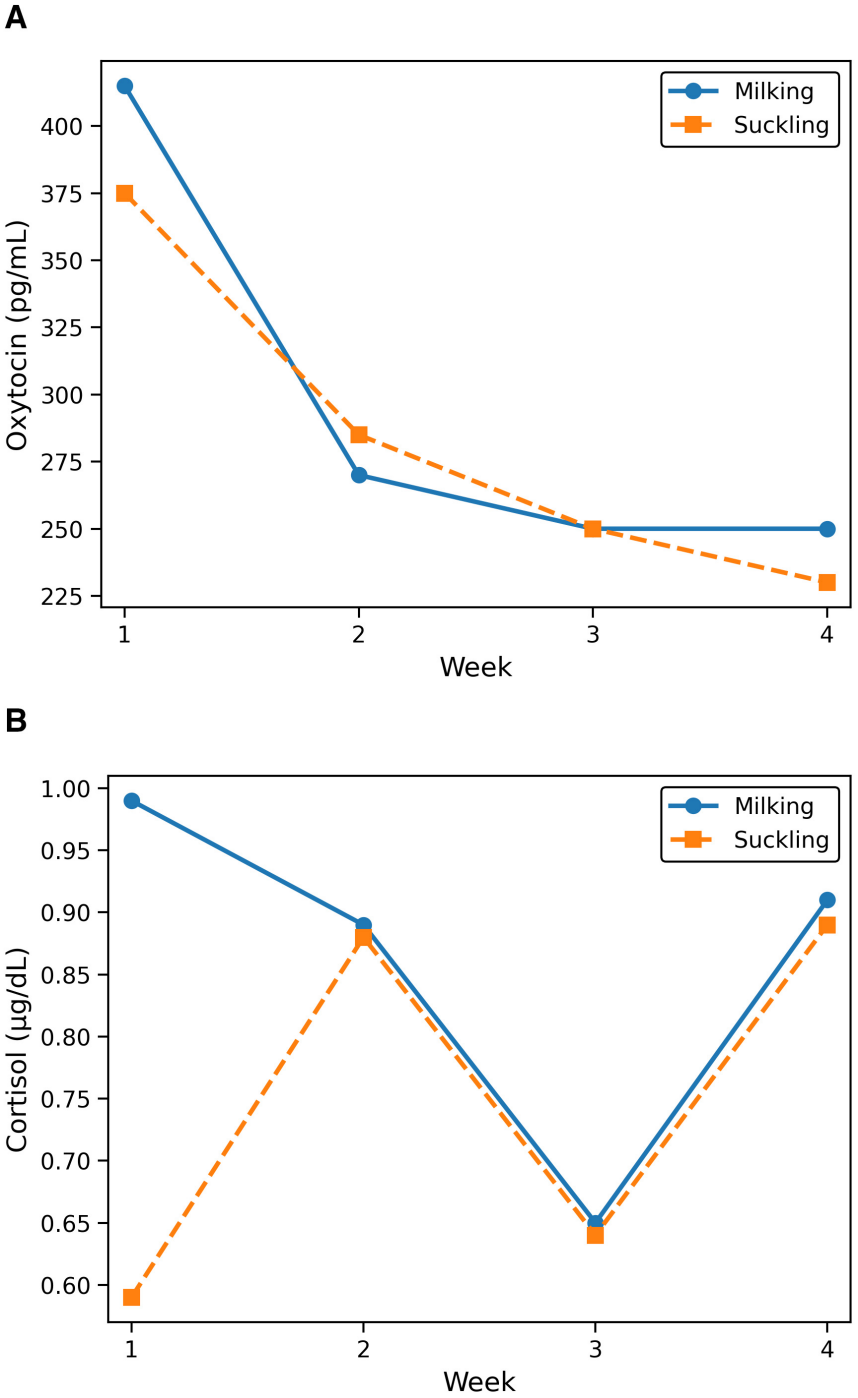
Weekly mean milk hormone concentrations in dairy goats (*n* = 20) across four consecutive weeks: **(a)** oxytocin (OT, pg/ml) and **(b)** cortisol (CORT, μg/dl). Values are shown for machine milking (solid line, circles) and kid suckling (dashed line, squares).

OT concentrations were highest in week 1 and declined over the subsequent weeks in both sampling contexts (Figure 1). During milking, mean OT decreased from 413.9 ± 168.7 pg/mL in week 1 to 250.8 ± 71.8 pg/mL in week 4. A similar pattern was observed during suckling (week 1: 374.2 ± 130.4 pg/ml; week 4: 230.3 ± 67.6 pg/ml). In contrast, CORT concentrations varied between weeks without a consistent temporal trend.

### 3.3 Housing conditions

Across all weeks and sampling contexts, does housed in the LD pen (greater space allowance) showed numerically higher mean OT concentrations than does in the HD pen (milking: 336 ± 141 vs. 257 ± 116 pg/ml; suckling: 309 ± 123 vs. 260 ± 111 pg/ml). Mean CORT concentrations were numerically lower in LD compared to HD (milking: 0.72 ± 0.63 vs. 1.00 ± 0.77 μg/dl; suckling: 0.64 ± 0.62 vs. 0.86 ± 0.76 μg/dl).

### 3.4 Associations with litter size and kid body weight

**Figure 2:**
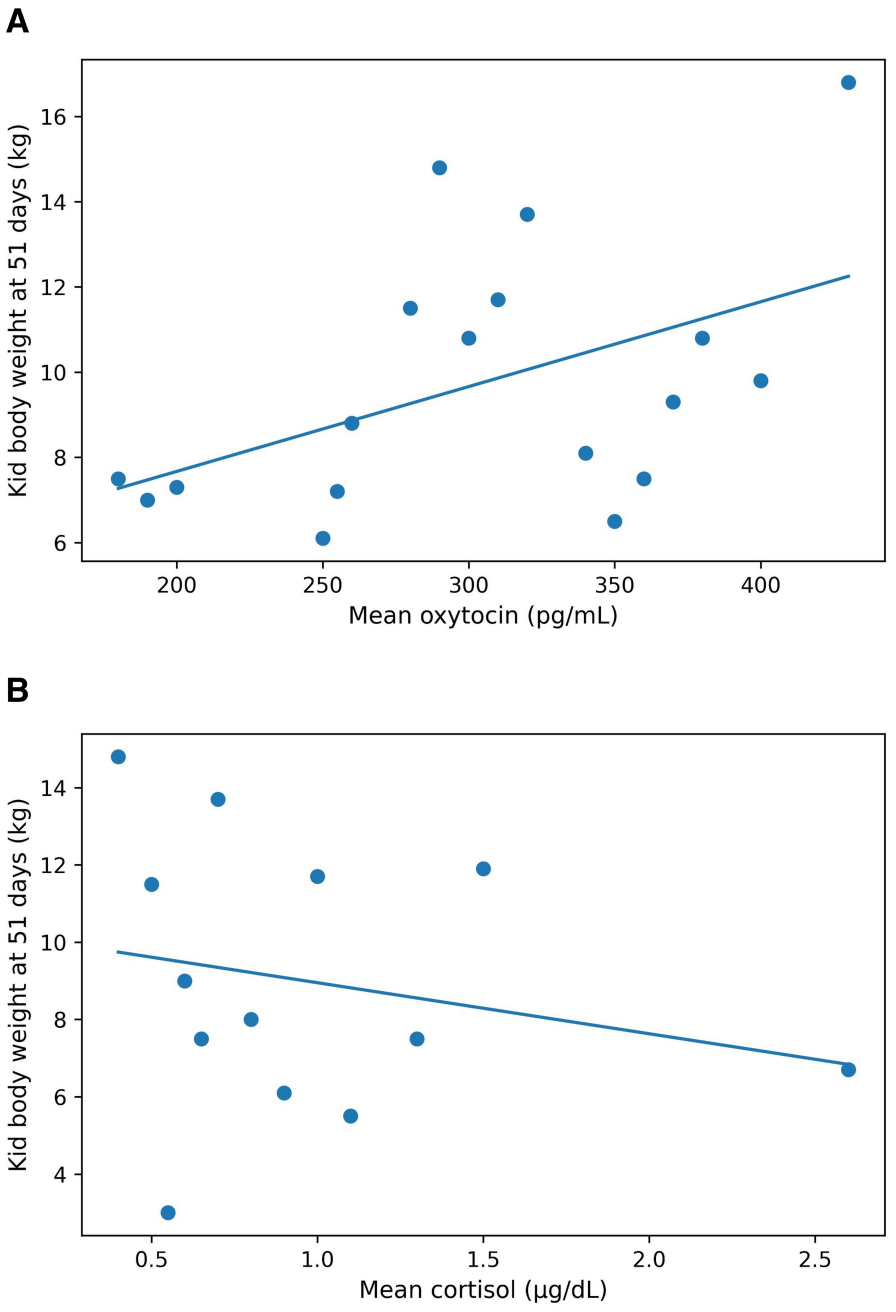
Descriptive associations between mean maternal milk hormone concentrations and kid body weight at 51 days of age: **(a)** oxytocin (OT, pg/ml) and **(b)** cortisol (CORT, μg/dl). Lines represent linear regression models fitted at the doe level.

Does rearing two kids showed higher mean CORT concentrations (1.04 ± 0.84 μg/dl) and lower kid body weight at 51 days (8.28 ± 2.25 kg) compared with does rearing single kids (0.53 ± 0.21 μg/dl; 11.49 ± 2.69 kg). Mean maternal OT showed a positive linear trend with kid body weight, whereas CORT showed a negative trend (Figure 2). When explored within housing conditions, associations were descriptively stronger in the HD pen (RMSE = 1.55 kg) than in the LD pen (RMSE = 2.59 kg).

## 4 Discussion

A novel aspect of this study is the quantification of OT and CORT in caprine milk under commercial conditions, a biological matrix that has received limited attention in relation to oxytocin and social environment in goats. OT concentrations were highest in the first week postpartum and declined thereafter, consistent with the well-established role of OT in milk ejection and early maternal activation (4–6). In contrast, the absence of a consistent temporal pattern in CORT concentrations may reflect its sensitivity to short-term environmental and physiological fluctuations rather than structured changes across early lactation. In addition to its role in lactation, OT contributes to maternal behavior and stress modulation (7, 8), supporting interpretation of early lactational peaks as part of broader maternal neuroendocrine adjustment.

As a first general observation, the milk OT and CORT concentrations observed in this study fall within biologically plausible ranges under farm conditions, supporting the validity of the measurements. When considering the two sampling contexts, milk OT concentrations were comparable between machine milking and kid suckling, aligning with earlier findings in goats that both stimuli elicit OT release (15). In contrast to studies in dairy cows reporting stronger OT release during suckling than machine milking (16), our results suggest similar endocrine responses across contexts. As does received concentrate during milking, it remains possible that feed-associated stimulation influenced OT secretion. The absence of marked differences in CORT between contexts further suggests habituation to routine handling, as also reported in small ruminants (17).

Does housed with greater space allowance showed numerically higher OT and lower CORT concentrations. Although housing conditions were not replicated, this pattern is consistent with evidence that stocking density and social stability influence stress physiology in livestock (9, 18). Within a Positive Animal Welfare framework, OT has been proposed as a candidate physiological indicator when interpreted alongside glucocorticoids (2, 10). Importantly, OT mobilization can occur in both positive and challenging contexts and may reflect adaptive social coping rather than simple positive valence (19, 20). Therefore, the observed housing-related differences may reflect variation in adaptive regulation rather than affective state *per se*.

Marked inter-individual variation in milk OT and CORT was observed, consistent with literature emphasizing contextual and individual modulation of OT-related processes (21). Such variability may reflect differences in temperament, social position, or metabolic allocation, although these factors were not directly measured.

Litter size was descriptively associated with maternal CORT concentrations and kid growth. Does rearing twins showed higher mean CORT and lower kid body weight at 51 days, consistent with increased energetic demands associated with multiple offspring (22). Early lactation imposes substantial metabolic load, and endocrine differences may reflect trade-offs in maternal investment (23). Regression analyses indicated positive associations between maternal OT and kid growth and negative associations between maternal CORT and kid growth. Comparable associations between milk hormones and offspring development have been reported in rodents (11), humans (12, 24), and non-human primates (13), although mechanisms in goats remain to be clarified. Hypotheses regarding hormone transfer from milk to offspring physiology require targeted investigation.

Several limitations must be acknowledged. Housing treatments were represented by single pens, precluding replication. Sample size was limited. OT and CORT assays were not fully validated for goat milk, and quantification of OT in complex matrices remains methodologically challenging (10). Behavioral measures and additional welfare indicators were not included. The absence of extraction procedures and matrix validation (e.g., parallelism and spike–recovery tests) limits certainty regarding assay accuracy in goat milk (10, 25). Consequently, findings should be interpreted as exploratory and hypothesis-generating.

Despite these limitations, the study demonstrates the feasibility of repeated milk-based measurement of OT and CORT in dairy goats under commercial conditions and suggests that these measures capture variation related to housing conditions and maternal physiological state. Further studies are needed to confirm these patterns in larger and replicated designs and to establish the analytical and biological validity of these measures, particularly within the framework of positive animal welfare.

## Data Availability

The raw data supporting the conclusions of this article will be made available by the authors, without undue reservation.
